# Design and development of a chimeric vaccine candidate against zoonotic hepatitis E and foot-and-mouth disease

**DOI:** 10.1186/s12934-020-01394-1

**Published:** 2020-07-11

**Authors:** Nouredine Behloul, Sarra Baha, Zhenzhen Liu, Wenjuan Wei, Yuanyuan Zhu, Yuliang Rao, Ruihua Shi, Jihong Meng

**Affiliations:** 1 College of Basic Medicine, Shanghai University of Medicine & Health Sciences, 279 Zhouzhu Highway, Pudong New Area, Shanghai, 201318 China; 2 Department of Gastroenterology, Zhongda Hospital, Southeast University, 87 Dijiaqiao Road, Nanjing, Jiangsu Province 210009 China; 3 China Institute of Veterinary Drug Control, Beijing, China

**Keywords:** Hepatitis E virus, Foot-and-mouth disease virus, Vaccine design, Chimeric vaccine, Bioinformatics, Immunogenicity

## Abstract

**Background:**

Zoonotic hepatitis E virus (HEV) infection emerged as a serious threat in the industrialized countries. The aim of this study is exploring a new approach for the control of zoonotic HEV in its main host (swine) through the design and development of an economically interesting chimeric vaccine against HEV and against a devastating swine infection: the foot-and-mouth disease virus (FMDV) infection.

**Results:**

First, we adopted a computational approach for rational and effective screening of the different HEV-FMDV chimeric proteins. Next, we further expressed and purified the selected chimeric immunogens in *Escherichia coli* (*E. coli*) using molecular cloning techniques. Finally, we assessed the antigenicity and immunogenicity profiles of the chimeric vaccine candidates. Following this methodology, we designed and successfully produced an HEV-FMDV chimeric vaccine candidate (Seq 8-P222) that was highly over-expressed in *E. coli* as a soluble protein and could self-assemble into virus-like particles. Moreover, the vaccine candidate was thermo-stable and exhibited optimal antigenicity and immunogenicity properties.

**Conclusion:**

This study provides new insights into the vaccine development technology by using bioinformatics for the selection of the best candidates from larger sets prior to experimentation. It also presents the first HEV-FMDV chimeric protein produced in *E. coli* as a promising chimeric vaccine candidate that could participate in reducing the transmission of zoonotic HEV to humans while preventing the highly contagious foot-and-mouth disease in swine.

## Background

Hepatitis E virus (HEV) is a serious health problem that threatens human lives worldwide [1]. Earlier, HEV (mainly genotypes 1 and 2) was considered as a threatening pathogen only in developing countries, where the virus is generally disseminated through contaminated water sources due to poor sanitation conditions [2]. Recently, however, the emergence of HEV infections (genotypes 3 and 4) is well recognized in the industrialized countries, with a zoonotic transmission route [3–5]. Continuous efforts have been directed towards the development of an effective strategy for the control and prevention of HEV infection [6]. In China, two recombinant vaccines based on human-HEV sequences have undergone clinical trials: HEV p239 (aa368–606 of ORF2 protein), which is already commercialized in China (Hecolin^®^) [7]; and the second is a virus-like particle (VLP) vaccine, HEV p179 (439-617aa of ORF2 protein), which is derived from HEV genotype 4 and expressed in *Escherichia coli* (*E. coli*) [8]. However, World Health Organization does not recommend the introduction of the HEV vaccine in national immunization programs except in special situations such as outbreaks where the risk of hepatitis E or its complications/mortality is particularly high [9]. With these measures, only the HEV genotypes 1 and 2 infections would be targeted in those special cases, while the zoonotic hepatitis E (genotypes 3 and 4) would continue to circulate in the non-endemic areas, not because the vaccine is ineffective against zoonotic genotypes but only because the use of hepatitis E vaccine is not implemented in the immunization programs in these non-endemic regions.

The control of nonhuman viral reservoirs is a worth investigating approach for the control of viral infection in humans. The most notable illustration is the effort for the control and the eradication of rabies through the removal of stray dogs, quarantine of incoming pets, and most importantly vaccination of domestic animals [10]. Likewise, this same approach would be of great public health interest, concerning zoonotic HEV infection. Since swine is the main host of zoonotic HEV (genotype 3 and 4) [3, 5], the control of HEV in swine will permit the prevention of HEV spread to humans. However, when it occurs in swine, hepatitis E infection is usually asymptomatic and self-limiting. Hence, the development of a swine vaccine that protects only against HEV would be of little interest to the swine breeding industry and most farmers will not use it because of its low benefit/cost ratio. Therefore, the development of a combined vaccine with bivalent protection against HEV and another swine virus, that causes severe disease, will increase the benefit/cost ratio and allow double protection. Foot-and-mouth disease virus (FMDV) stands as one of the best targets for such a combined vaccine, and this for two major reasons: (1) FMDV is the causative agent of a highly infectious disease of cloven-hoofed animals. The infection spreads rapidly through susceptible animal populations and can lead to large-scale epidemics with detrimental economic consequences [11]. (2) Given the severity of the FMDV infection and its outcomes, vaccination against FMDV is mandatory in China, among many other countries [11].

Therefore, taken the above-mentioned facts, we aim to investigate and produce an HEV-FMDV chimeric vaccine candidate in *E. coli*, that would allow a double protection: on one hand, against the FMDV infection that causes heavy and severe implications for swine farming; and on the other hand, against HEV infection which would permit an effective control of its zoonotic transmission to humans.

## Results

### Design, properties and 3D structure models of the chimeric proteins

To ensure a broad protectivity, the FMDV antigen Seq 8 was designed by combining three entire VP1 G–H loops of three FMDV strains (O/Mya/98, O/HN/CHA/09, and O/IRN/2010). Four HEV ORF2-truncated proteins p166 (aa452–617), p179 (aa439–617), p216 (aa420–637) and p222 (aa439–660) comprising the dominant neutralizing epitopes of the HEV capsid protein were selected to be combined with the FMDV antigen. The chimeric antigens were then designed by attaching the HEV fragment to the N- or C-terminal ends of Seq 8. Accordingly, a total of 8 HEV-FMDV combined antigens have been designed. The 3D structure models of these combined proteins and individual antigens (namely: P166, p179, P216, P222, and Seq 8) have been predicted, refined and evaluated. The best model for each protein (see Additional files 1, 2) has been selected for further analysis.

### Antigenicity analysis by computational methods

**Fig. 1:**
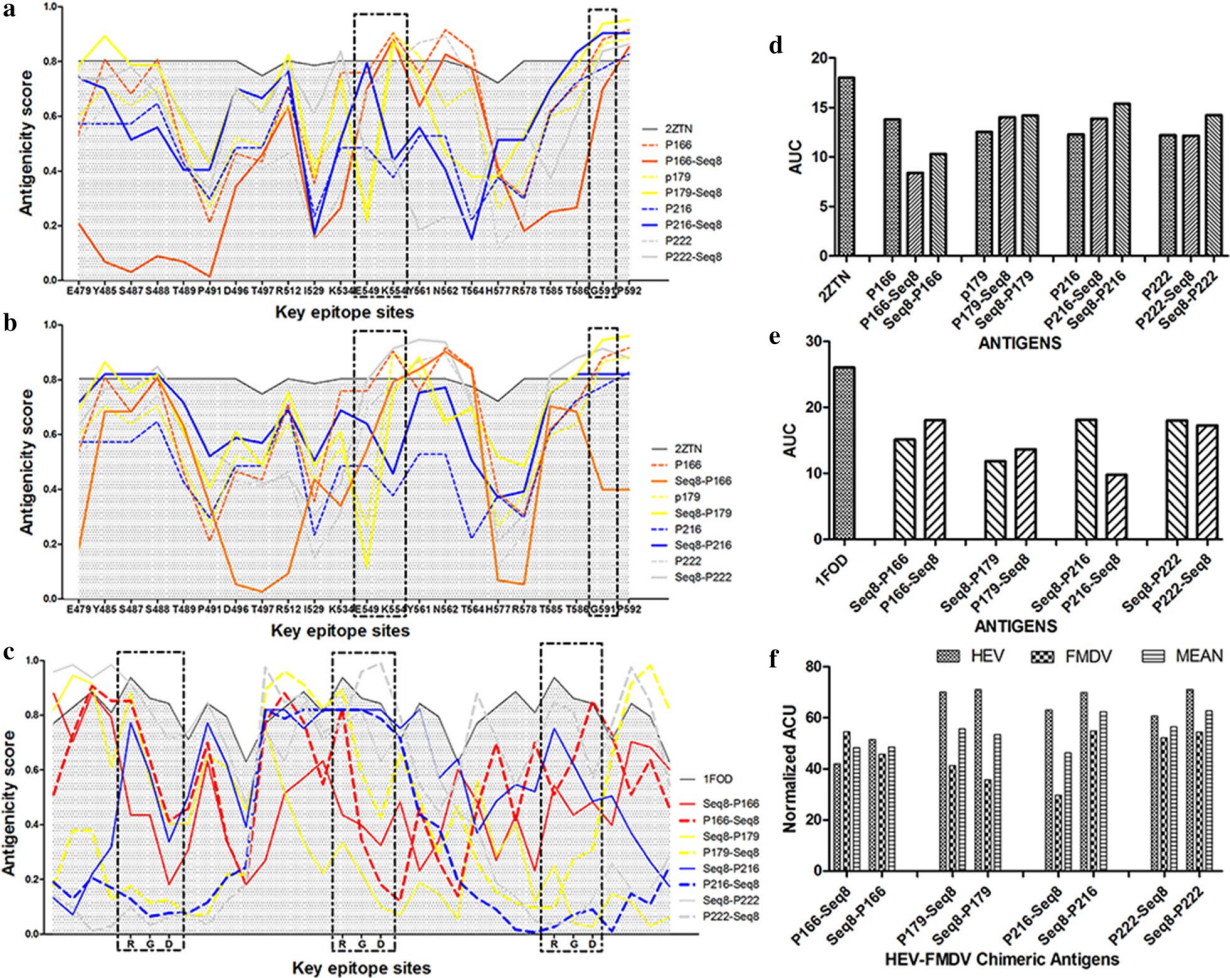
Protrusion index of key-epitope sites of HEV and FMDV antigens within the HEV-FMDV chimeric proteins as calculated by the Ellipro web server. This figure shows the protrusion index of the key-epitope sites: **a** in the HEV antigens alone and when they are attached to the N-terminal of the FMDV Seq 8 antigen; **b** in the HEV antigens alone and when they are attached to the C-terminal of the FMDV Seq 8 antigen; and **c** in the FMDV Seq 8 antigen when it is attached to the N-terminal or C-terminal of the different HEV antigens. Next, the total areas under the curves of the protrusion indexes were calculated for each protein using GraphPadPrizm5 Software (**d**–**f**) and are shown in **d** for the HEV antigens alone and when they are attached to either the N- or C-terminal of the FMDV Seq 8 antigen; and in **e** for the FMDV Seq 8 antigen when it is attached to either the N- or C-terminal of the HEV antigens. The normalized AUCs of the protrusion indexes of the key epitopes for both HEV and FMDV fragments in the different chimeric proteins are summarized in **f**

The protrusion from the protein surface of the selected key-epitope sites was calculated by the Ellipro web server. The results revealed that, as expected, the protrusion index (PI) of the different HEV and FMDV key sites varied greatly between the different combinations (Fig. 1a–c). Therefore, to better appreciate the overall antigenicity and to make it easier to select the best combinations, the total area under the protrusion index curve (AUC) of each protein was calculated and plotted as bar charts. Since the protrusion index data tables have no X values, the area-under-curve was computed using row numbers as X. Therefore, since the number of the selected key epitopes differs between HEV and FMDV viruses, the AUCs were normalized to the maximum score as follows: the normalized AUC = 100*(Calculated AUC/reference AUC). The reference AUC for a given set of key residues equals the number of selected key residues multiplied by one (where one is the maximum protrusion index for a fully exposed residue). A higher normalized AUC indicates a higher exposition of the selected key epitopes, which in turn suggests that the epitopes are less affected by the combination with the other antigen.

When compared to the reference HEV structure 2ZTN, the overall antigenicity of the chimeric proteins was found slightly lower (Fig. 1d). However, the 2ZTN structure was determined using the ORF2 protein of an HEV genotype 1 strain, while all the chimeric proteins were designed according to the capsid protein of an HEV genotype 4 strain. Therefore, given the difference in amino acid sequences of the ORF2 protein between the two strains, it was expected that the expositions of the antigenic entities would differ. On the other hand, when the chimeric proteins were compared to the individual HEV antigens also designed from a genotype 4 strain (P166, P179, P216, and P222), the overall antigenicity of the HEV moieties increased by the addition of the Seq 8 antigen, except when this latter is added to the P166 protein (Fig. 1d). It was also noted that placing the FMDV Seq 8 antigen at either the N- or C-terminal of P179, P216 and P222 did not affect the exposition of the HEV key epitopes, but when combined with P166 both combinations reduced the epitopes exposition (Fig. 1a, b, d). Concerning the FMDV key epitopes, they were more exposed when Seq 8 was attached to either side of P222 and P166. While with P216, placing Seq 8 at the N-terminal offered a better exposition of the epitopes (Fig. 1c, e).

Overall, by analyzing the normalized AUCs (Fig. 1f), two combinations Seq 8-P222 and Seq 8-P216 exhibited the best exposition of the key-epitope sites of both HEV and FMDV (normalized AUCs of 71.1–54.5 and 69.9–54.9 respectively). Therefore, these two chimeric proteins have been chosen as the best candidates for the HEV-FMDV combined vaccine.

### Flexibility of the chimeric proteins

The Seq 8-P216 and Seq 8-P222 have shown the best exposition scores of both HEV and FMDV epitopes. Next, the CABS-flex server was used to perform molecular dynamics simulation to analyze the flexibility of these selected chimeric proteins and its effects on the epitopes sites presentation.

**Fig. 2:**
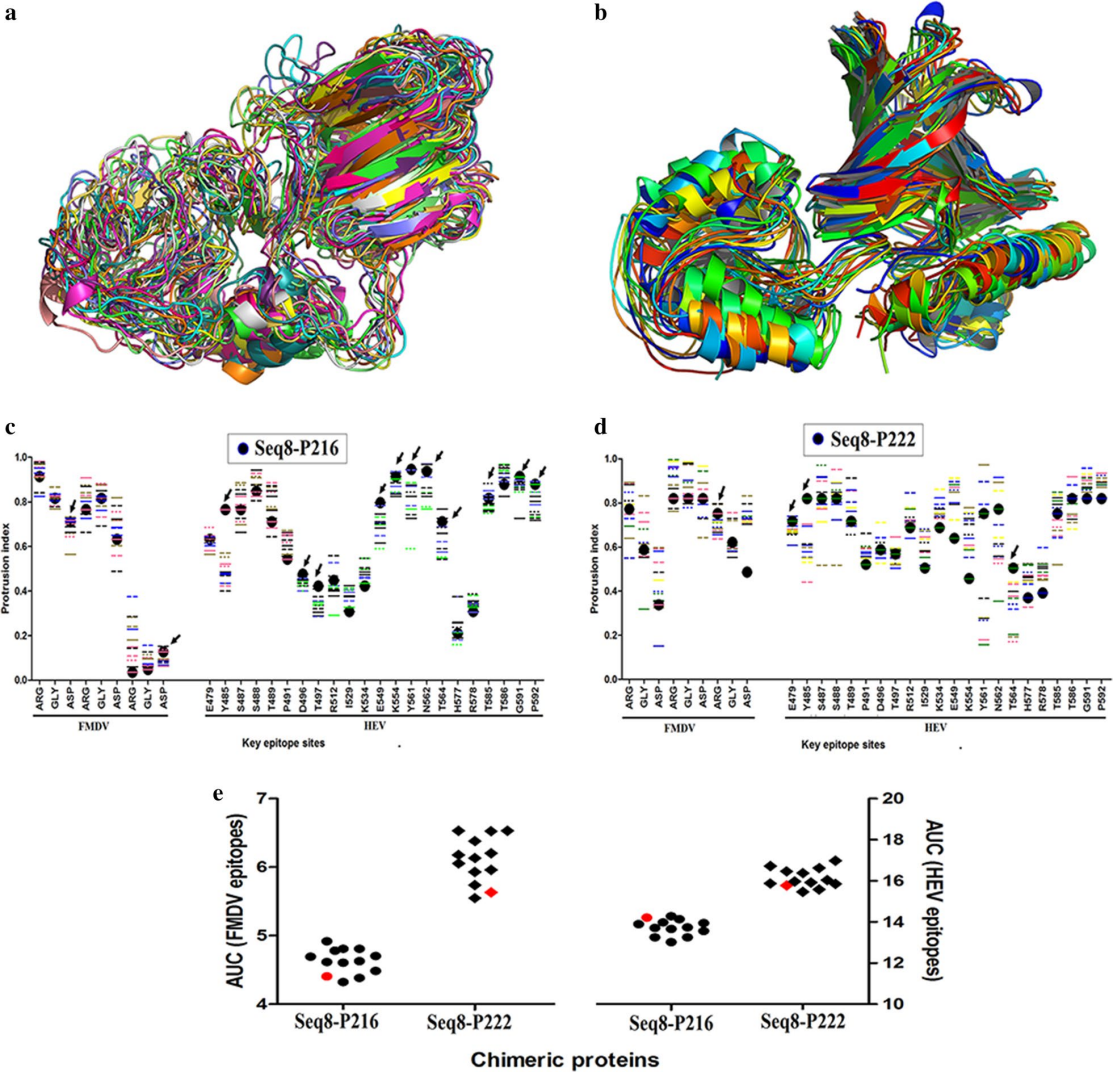
Prediction of the flexibility of HEV-FMDV chimeric proteins. The different conformational models of Seq 8-P216 (**a**) and Seq 8-P222 (**b**) predicted by the CABS-flex server were superimposed and visualized by Pymol. The fluctuations of the protrusion index of the key epitope sites between the different conformations were calculated for both Seq 8-P216 (**c**) and Seq 8-P222 (**d**): the black circles represent the PI of the epitope sites in the input models; the lines represent the PI for the same residues in the 12 models predicted by the molecular dynamics simulation; the black arrows indicate the residues with a PI decrease in most of the 12 models compared to the input model. The overall exposure of HEV and FMDV epitopes in Seq 8-P216 and Seq 8-P222 chimeric proteins were computed for each model (**e**): the exposure is represented as the area under the curve of the protrusion index of the key-epitope sites (the method is detailed in the “Antigenicity analysis” section); circles and diamonds in red represent the overall antigenicity of the initial models; circles and diamonds in black represent the overall antigenicity of different models predicted in the molecular dynamics simulations

The server performs the 10 ns simulation and analyses 2000 conformations of the input structure, and then it clusters these conformations into 12 clusters according to the similarity of the models based on the Cα root-mean-square deviation (RMSD) value. The results showed that the different conformation clusters calculated for Seq 8-P216 and Seq 8-P222 exhibited a Cα RMSD average of 2.03 ± 0.14 and 1.79 ± 0.11, respectively (see Table S1 in Additional file 3). Further, the fluctuations between the different clusters of Seq 8-P216 and Seq 8-P222 conformations were calculated in terms of Cα RMSD and Cα GDT-TS (Global Distance Test-Total Score). The results of both metrics (see Table S2 in Additional file 3) indicated that Seq 8-P222 models share more similarity than the Seq 8-P216 models and therefore this latter would show more mobility in an aqueous solution. In other terms, the Seq 8-P216 was more flexible than Seq 8-P222 and this flexibility was more visible by the superimposition of the predicted models as shown in Fig. 2a, b, where the fluctuation was more important in Seq 8-P216, especially in the Seq 8 subunit.

Next, the representative models of the 12 clusters were used to calculate the protrusion index of HEV and FMDV key-epitope sites in the chimeric proteins as detailed in the previous section (antigenicity analysis). Figure 2c, d show the fluctuations of the PI of the key-epitope residues between the different conformations of Seq 8-P216 and Seq 8-P222. The PI of several HEV key epitopes (black arrows) decreased in most Seq 8-P216 conformations compared to the initial model used in the antigenicity analysis; two out of the nine FMDV key sites were also found less exposed in most Seq 8-P216 models. Concerning Seq 8-P222, only 3 HEV epitopes and one FMDV epitope exhibited lower PI in the different conformations than in the input model. This indicates that the flexibility of Seq 8-P222 in aqueous solution does not affect much the exposure of HEV and FMDV epitopes, and even increases their protrusion in most conformations (Fig. 2e). Meanwhile, the mobility of Seq 8-P216 seems to especially reduce the antigenicity of the P216 subunits, which would affect the overall antigenicity of the chimeric protein.

### Plasmid construction and protein expression and purification

**Fig. 3:**
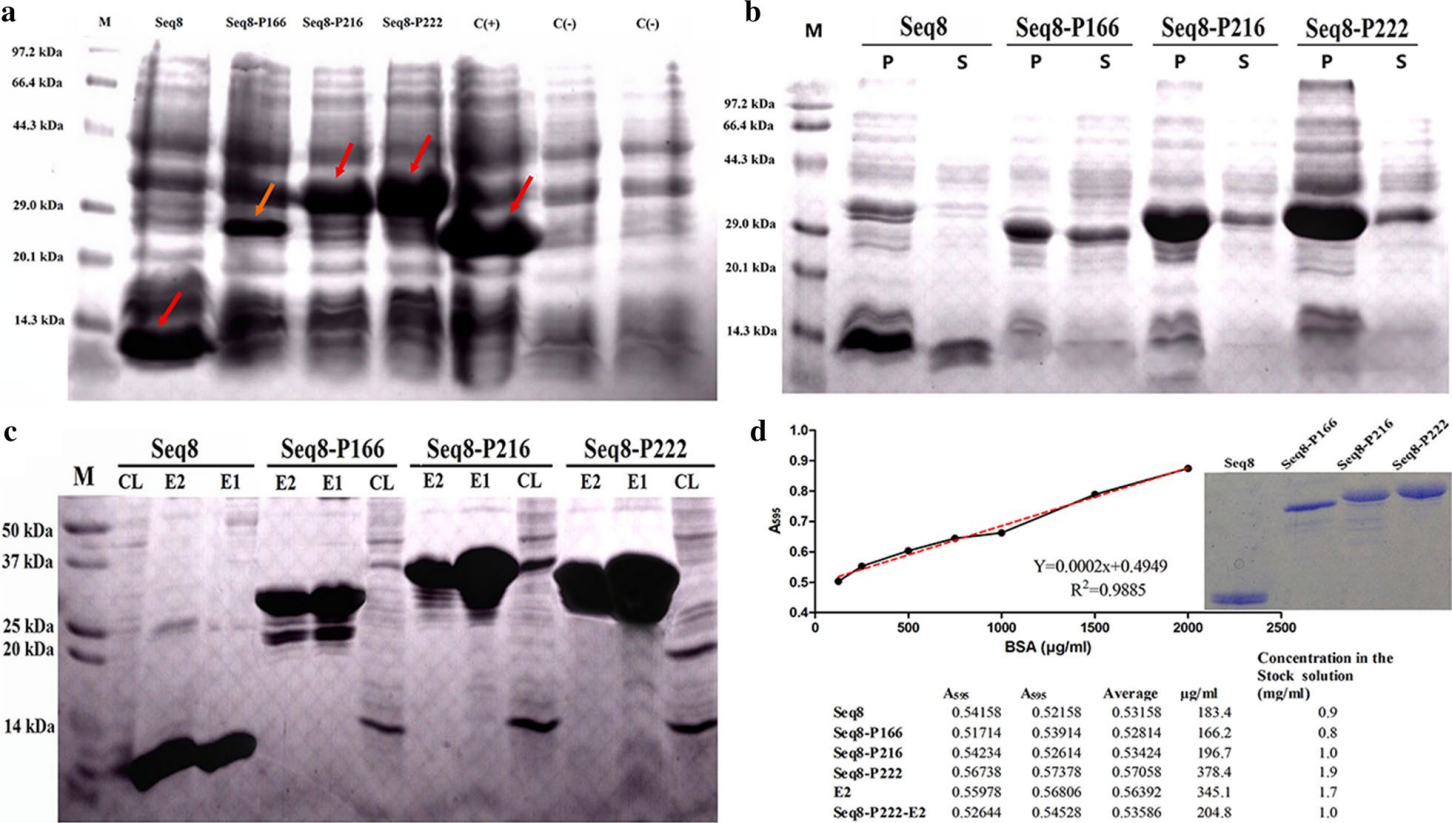
Expression and purification of the HEV-FMDV chimeric proteins. **a** A 15% SDS-PAGE gel showing the overexpression of Seq 8, Seq 8-P166, Seq 8-P216 and Seq 8-P222 at the expected molecular weights of 9.4, 27.4, 33.4 and 33.9 kDa respectively; and no extra-bands are visible before the IPTG induction (negative control). M: molecular weight marker; C (−): negative control, before IPTG induction; C (+): induction of P222 expression as a positive control. **b** SDS-PAGE analysis of the solubility of the expressed proteins; P: pellet (insoluble fraction); S: supernatant (soluble fraction). **c** SDS-PAGE analysis of the purified FMDV antigen and HEV-FMDV chimeric proteins; M: molecular weight marker; CL: cell lysates after passing through the Ni–NTA agarose column; E1 and E2: elution 1 and elution 2 respectively. **d** Determination of the protein concentrations using the Bradford protein assay: the protein concentration was calculated in the diluted samples then multiplied by 5 (dilution factor) to determine the concentrations in the stock solutions

The target genes have been successfully amplified, purified and inserted into the pET28a (+) vector. Next, competent *E. coli* BL21 (DE3) cells were successfully transformed using the expression constructs. After the induction of expression using IPTG, the FMDV Seq 8 antigen and the HEV-FMDV combined proteins were highly over-expressed in *E. coli* as shown in Fig. 3a. The Seq 8-P222 combined protein showed the highest expression level followed by Seq 8 and Seq 8-P216 while Seq 8-P166 was less expressed than the other proteins. The solubility analysis revealed that the four recombinant proteins were obtained in both soluble and insoluble fractions (Fig. 3b) and there were enough proteins in the soluble fractions to proceed with the purification under native conditions (Fig. 3c). Next, the purified proteins were diluted 5 times and by adopting the Bradford assay approach the protein concentrations were determined to range from 0.8 mg/ml for Seq 8-P166 to 1.9 mg/ml for Seq 8-P222 as shown in Fig. 3d. It is to note that approximately 2 ml of purified proteins were obtained for each one of the target proteins from 200 ml of bacterial culture, indicating thus a relatively high yield.

### Stability analysis of FMDV and HEV-FMDV recombinant proteins

**Fig. 4:**
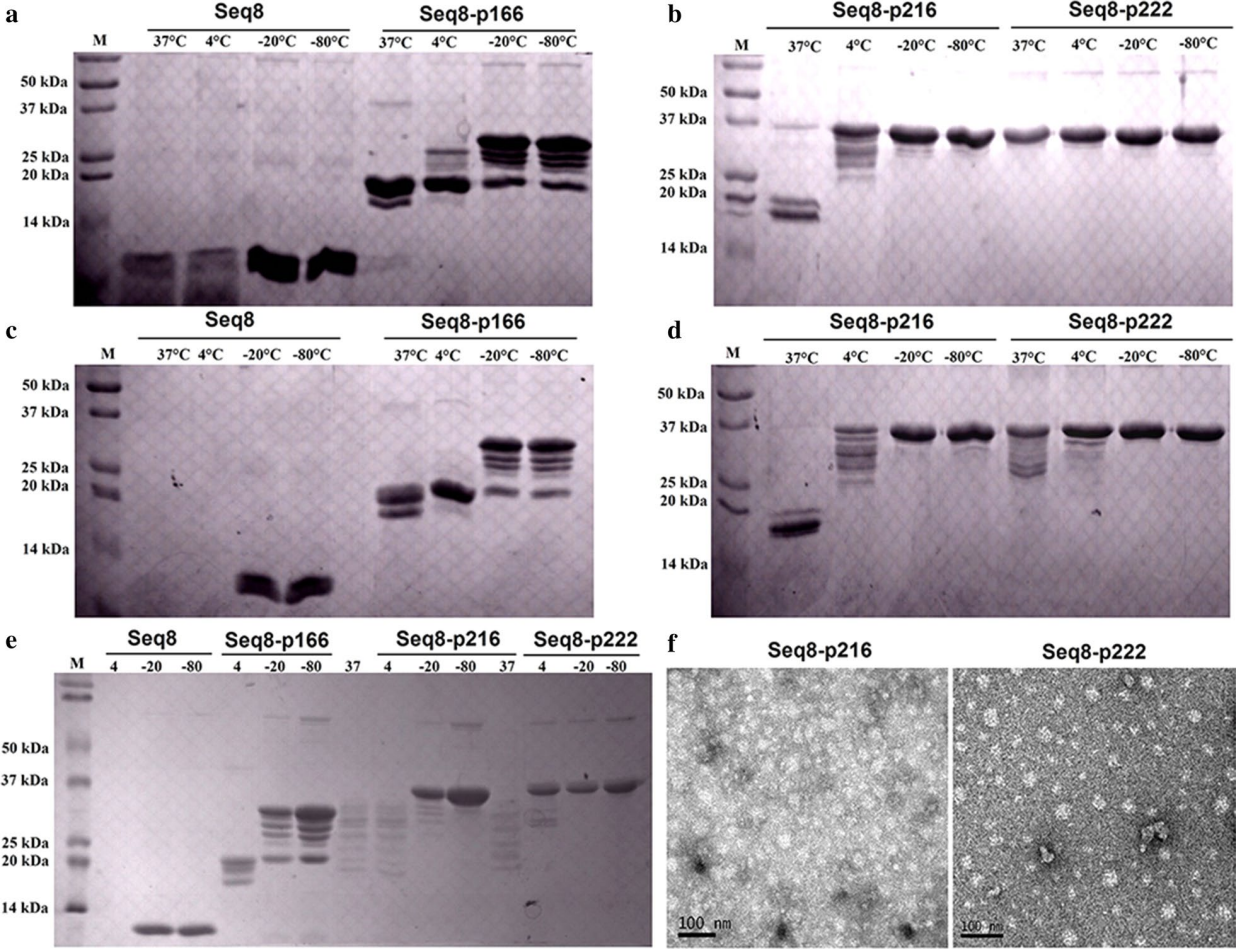
Stability of FMDV and HEV-FMDV recombinant proteins stored at different temperatures and analyzed by SDS-PAGE after 2 weeks (**a**, **b**), 6 weeks (**c**, **d**) and 10 weeks (**e**). **f** Electron micrographs of Seq 8-P216 and Seq 8-P222 VLPs generated in *E. coli*; the VLPs were negatively stained with 2% uranyl acetate; bar: 100 nm

The thermal-stability analysis results are shown in Fig. 4. All the proteins were stable at − 20 and − 80 °C throughout the 10 weeks of the experiment. After 2 weeks (Fig. 4a, b), at 37 and 4 °C, only Seq 8-P222 was stable while the Seq 8 and Seq 8-P166 antigens were degraded at both temperatures. It is worth noting that the degradation of Seq 8-P166 yielded a fragment of about 18 kDa, which corresponds to the molecular weight of P166 alone. For Seq 8-P216, the degradation was quasi-complete at 37 °C; and at 4 °C, only a small fraction of the protein was degraded. After 6 weeks (Fig. 4c, d), Seq 8 was fully degraded at 37 °C and 4 °C while for Seq 8-P166 no bands were visible at the expected molecular weight but the 18 kDa degradation product remained stable. Likewise, the Seq 8-P216 stored at 37 °C was fully degraded and also yielded a ~ 18 kDa stable fragment while at 4 °C only a small fraction was still visible at the expected position. Although signs of degradation were visible at 37 and 4 °C, strong Seq 8-P222 bands remained visible. After 10 weeks (Fig. 4e), the Seq 8-P216 proteins stored at 37 °C and 4 °C were completely degraded and this same observation was also noted for Seq 8-P166 stored at 4 °C. Meanwhile, the proteolysis of Seq 8-P222 stored at 4 °C did not increase between the 6th and the 10th week and a strong band was still visible at the expected position.

### Transmission electron microscopy

The visualization of the combined proteins using the transmission electron microscopy, revealed that both Seq 8-P216 and Seq 8-P222 could self-assemble into virus likes particles of different diameters (up to ~ 40 nm) as shown in Fig. 4f. Meanwhile, Seq 8 seems to form small aggregates of less than 10 nm in diameter but no clear particles, comparable to those of the combined proteins, were visible on the Seq 8 micrograph (Data not shown).

### Antigenicity of the HEV-FMDV chimeric proteins

**Fig. 5:**
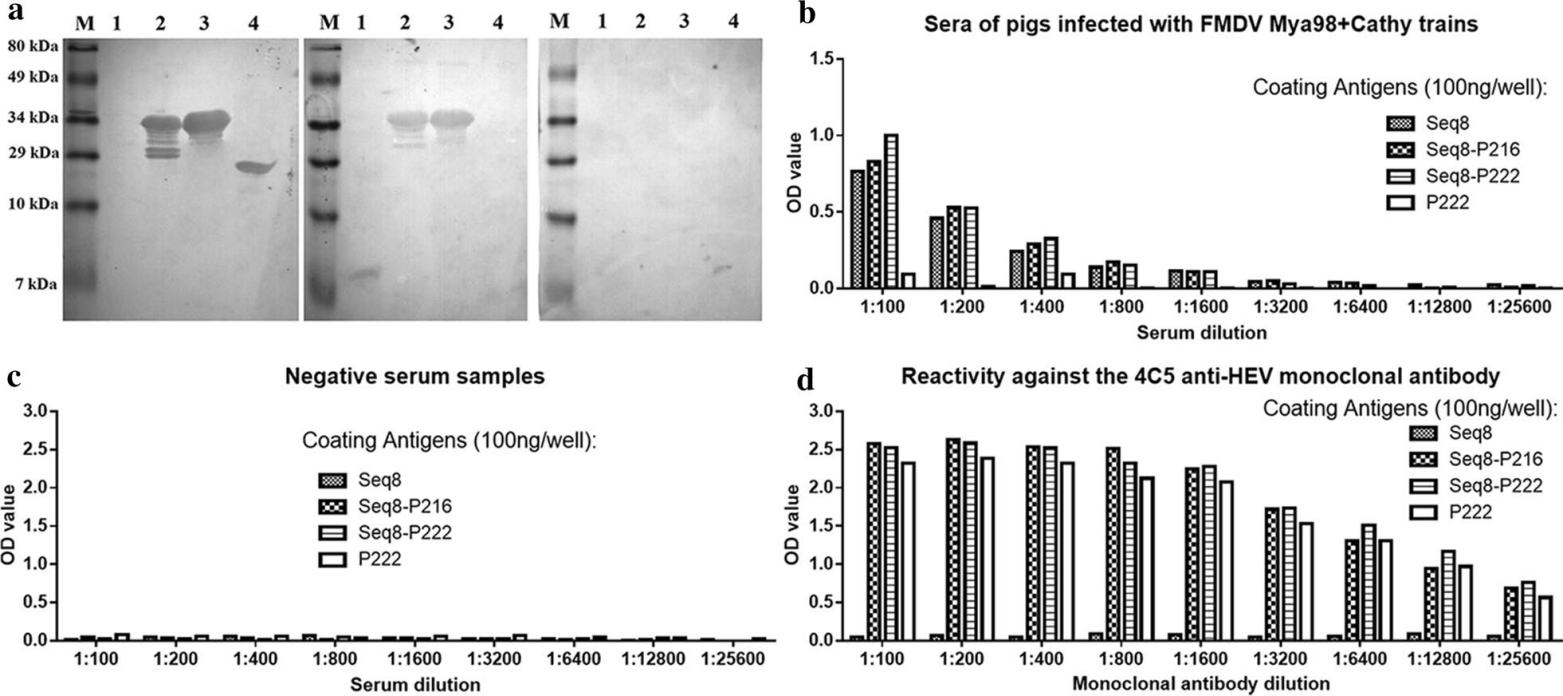
Antigenicity of the HEV-FMDV chimeric proteins. **a** Western blot analysis using anti-HEV 5G5 monoclonal antibody (left), commercial porcine polyclonal antibodies used against FMD (middle) and serum of mice free of anti-HEV and anti-FMDV antibodies (right); M: molecular weight marker; lanes 1-4: Seq 8, Seq 8-P216, Seq 8-P222 and P222, respectively. **b** Assessment by indirect ELISA of the reactivity of the chimeric antigens against the anti-FMDV antibodies present in the sera of pigs infected with FMDV strains. **c** Assessment of non-specific reactivity of the chimeric antigens using mice sera free of anti-FMDV and anti-HEV antibodies. **d** Reactivity of the chimeric antigens against the HEV-neutralizing 5G5 monoclonal antibody

After the purification of the combined proteins, we sought to investigate whether these combined antigens could react against both anti-HEV and anti-FMDV antibodies. The Western blotting results revealed that indeed the Seq 8-P216 and Seq 8-P222 proteins could react against the 5G5 monoclonal antibody (Fig. 5a, left) and against the anti-FMDV polyclonal antibodies (Fig. 5a, middle). Meanwhile, the P222 protein reacted only against the 5G5 antibody and Seq 8 reacted only against the anti-FMDV antibodies. This indicated that the HEV and FMDV antigenic entities were well conserved in the combined proteins. Moreover, none of the blotted proteins reacted against the serum of mice free of anti-HEV and anti-FMDV antibodies (Fig. 5a, right). These results were further confirmed in the indirect ELISA experiments where Seq 8 and the two HEV-FMDV chimeric proteins reacted against the antibodies induced by the FMDV Mya98 and Cathy strains in pigs (Fig. 5b). It is worth noting that Seq 8-P222 was more reactive against the FMDV-specific antibodies than the Seq 8 alone which was consistent with the computational analysis results. To ensure that the reactivity observed was that of the Seq 8 subunit, the pig sera were tested using the HEV P222 alone as a coating antigen and the results were negative, indicating thus that the tested pig sera were free of anti-HEV antibodies and the reactivity observed with the chimeric protein was indeed that of Seq 8 entity. Furthermore, the chimeric proteins also reacted strongly against the HEV neutralizing 5G5 antibody, which indicates that the HEV neutralization epitopes were well conserved in the combined proteins (Fig. 5d).

### Immunogenicity of the HEV-FMDV chimeric proteins

Serum samples from all animals were tested for the presence of FMDV-specific antibodies to determine whether the immunization with the HEV-FMDV combined proteins can induce an antibody response comparable or distinguishable from that of the animals inoculated with the commercially available FMD vaccine. The sera were collected from the immunized mice at 2, 4, 6, 8 and 12 weeks after the first inoculation.

**Fig. 6:**
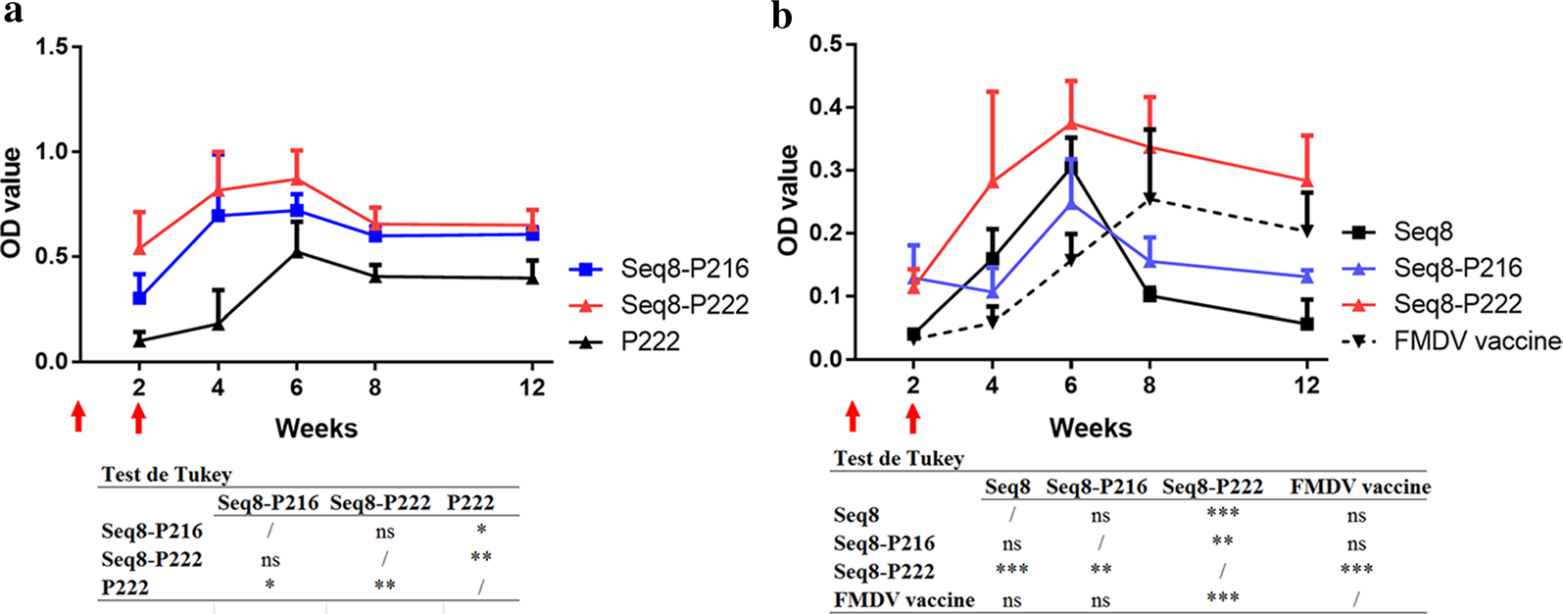
Immunogenicity of the HEV-FMDV chimeric proteins. This figure shows the levels of the HEV- and FMDV-specific antibodies in the sera of the immunized mice at different post-immunization time points (the sera were diluted 1:25,600). The different groups were analyzed by two-way ANOVA followed by Tukey’s test. **a** Titers of HEV-specific antibodies; **b** Titers of FMDV-specific antibodies; *P < 0.05, **P < 0.01; ***P < 0.001; ns: no significance, P > 0.05; red arrows: first and second immunizations

As shown in Fig. 6 and Additional file 4, all the vaccinated animals produced detectable FMDV-specific IgG antibodies 2 weeks after the first immunization. The reactivity of the antibodies increased in week 4 in the Seq 8, Seq 8-P216, and Seq-8P222 groups; while no change was observed in the FMD vaccine group. At week 2, the animals received the second dose of the immunogens. At week 6 (4 weeks after the second dose), the FMDV-specific IgG titers increased considerably in the four groups with the highest values observed in the Seq 8 group. However, this increase was not permanent in the Seq 8 and Seq 8-P216 groups where the antibodies titers decreased at week 8. The reactivity of the antibodies continued to decrease in week 12 for the Seq 8-P216 group but remained at the same level in the Seq 8 group. Meanwhile, for the FMDV vaccine and Seq 8-P222 groups, the antibodies titers remained unchanged at weeks 8 and 12 with a stronger reactivity in the Seq 8-P222 group as shown by the values of the last serum dilutions (1:25,600).

The sera were then tested by indirect ELISA for the presence of HEV-specific antibodies using the HEV P166 antigen as a detector. The results are shown in Fig. 6 and Additional file 5. The anti-HEV IgG antibodies were detected in all the immunized mice 2 weeks after the first immunization. As expected, antibody levels increased after the boost inoculations (week 2). Overall, the kinetics of antibody responses was similar in all the groups and all the immunized mice produced the same high levels of HEV-specific antibodies that remained almost unchanged until the end of the experiment (week 12). It is to note, however, that the antibody levels were higher in the Seq 8-P216 and Seq 8-P222 groups than in the P222 control group. This could be explained by the difference in the amount of the protein in the immunization dose (10 µg of P222 versus 50 µg of the chimeric proteins).

## Discussion

The selected HEV and FMDV fragments for the design of the HEV-FMDV chimeric vaccine have been extensively studied. Numerous reports demonstrated that these individual proteins could mimic the main antigenic characteristic of the corresponding viruses [8, 12–17]. However, combining two different fragments together would yield a new protein that might have different antigenicity. Therefore, to determine how HEV and FMDV proteins would affect each other, we designed 8 different combined proteins and analyzed them by various computational methods.

Analysis and prediction of three-dimensional structures of biological macromolecules such as proteins are among the prominent research areas in the computational biology field [18, 19]. Insights into the protein structural features allow a deeper investigation of its biological functions, in our case, its antigenic composition. Therefore, we first determined the 3-dimensional structures of the designed HEV-FMDV combined proteins using one of the latest generation strategies in structural bioinformatics known as fold recognition and threading method [20, 21]. After obtaining the best structural models, we sought to evaluate the difference between the HEV-FMDV chimeric proteins at a conformational level. The HEV epitopes are conformational epitopes that consist of sequential segments that are brought together in spatial proximity when the corresponding protein is folded [17, 22]. Furthermore, several key-epitope sites have been identified and reported to be crucial for eliciting HEV neutralizing antibodies [13–16]. Likewise, the whole G-H loop of the FMDV VP1 protein is believed to elicit FMDV neutralizing antibodies and the RGD domain plays a pivotal role in this process [23–25]. The evaluation of the exposition to the surrounding environment of these key residues (HEV and FMDV epitopes) revealed that two combined proteins (Seq 8-P216 and Seq 8-P222) could highly mimic the antigenicity of the individual HEV and FMDV antigens. This could be due to the extension at the N-terminal end of P216 and P222. In the HEV virus-like particles, the M and P domains are linked with a long proline-rich hinge (amino acids 445–467) and the flexibility of this hinge region allows the unique feature of HEV capsid where the P domain dimers protrude from the virus shell [26]. Likewise, this flexible extension in P216 and P222 could play a role in keeping the Seq 8 antigen far enough from the P domain of P216 or P222 and therefore, limit the effects of each other on the exposure of the key-epitope residues. Further, since proteins in aqueous solution are in constant mobility, we have to ensure that this mobility cannot affect the exposure of HEV and FMDV epitopes in the combined antigens. The molecular dynamics simulations revealed that the fluctuations of Seq 8-P222 improved the accessibility of the key-epitope sites and thus its overall antigenicity; while the antigenicity of Seq 8-P216 was negatively affected by its flexibility in the aqueous environment.

Although high-level expression of heterologous proteins in *E. coli* is still a challenge for many targeted heterologous genes [27, 28], the HEV-FMDV chimeric proteins were highly over-expressed. This has been also observed for several fragments of the HEV ORF2-encoded proteins that have been investigated over the years: p166 (aa452–617), p179 (aa439–617), p239 (aa368–606), E2s (aa454–606), aa112–607 and aa458–607 fragments [12, 17, 29, 30]. Likewise, the FMDV VP1 protein and its related fragments and chimeric proteins have been also reported to be well expressed in *E. coli* [31–33]. Combining the FMDV antigen to the HEV P216 and P222 proteins did not affect their good expressivity in *E. coli* and an important fraction of these chimeric proteins were soluble which permitted their purification under natural conditions using a simple and easy procedure. Moreover, the HEV-FMDV chimeric proteins were found able to self-assemble into VLPs, a feature that has gained considerable interest in the vaccine development field [34, 35]. Altogether, these results permitted making the first step towards the development of a cost-effective HEV-FMDV chimeric vaccine.

The results of the stability analysis indicated that the Seq 8-P222 was very stable a 37 °C for more than 6 weeks and at 4 °C for more than 10 weeks. In other words, Seq 8-P222 seems to be stable for more than 6 weeks without cold chain requirements and this represents a very attractive property for a vaccine. Moreover, the proteins were tested without any formulation and thus we could speculate that these periods could be extended with an adequate adjuvant formulation as reported previously [36, 37]. It is worth noting that the instability of Seq 8-P216 could be due to the fluctuations in the Seq 8 subunit predicted in the flexibility analysis (Fig. 2). However, further analysis of a larger set of proteins is needed to confirm this observation.

One of the major concerns for using the FMDV VP1-derived immunogens is the hypervariability of the immunodominant G–H loop domain. As it has been reported earlier, the amino acid substitutions in the G–H loop domain could alter the interaction of FMDV strains with the neutralizing antibodies [38]. However, despite this hypervariability, several studies have reported the protective effects of VP1-based vaccines [32, 33, 39–41]. Consequently, the Seq 8 antigen was designed to ensure a large spectrum of antigenicity by combining the entire G–H loop domains of three different FMDV strains that were responsible for major epidemics in the past, and still represent threats of future outbreaks in China.

The antigenicity experiments revealed that the HEV-FMDV chimeric proteins strongly reacted against the HEV neutralizing antibody in both the ELISA and Western blotting experiments, thus indicating that the exposition of the HEV conformational epitopes on both Seq 8-P216 and Seq 8-P222 chimeric proteins were not affected by the addition of the Seq 8 fragment. Likewise, the Seq 8 antigen alone or combined to HEV antigens reacted against the FMDV specific antibodies present in the sera of pigs infected with different FMDV strains, indicating that the antigenicity of the FMDV G–H loop domain is well conserved in Seq 8 antigen and the conformation of its antigenic entity was not altered in the chimeric proteins. Furthermore, upon injection into mice, the HEV-FMDV chimeric proteins elicited the production of almost similar levels of anti-HEV IgGs. However, the difference in the humoral immune response was significant concerning the anti-FMDV antibodies, with Seq 8-P222 being more immunogenic. This indicates that unlike P216, combining P222 with Seq 8 improved the immunogenicity of this latter antigen. However, determining the mechanism underlying this improvement needs further investigation.

One of the limitations of this immunogenicity study was the lack of FMDV neutralization assay. Because the FMD is one of the most contagious and deadly diseases of cloven-hoofed animals, special laboratory settings according to the recommendation of the World Organization for Animal Health (OIE) are required to perform neutralization assays using virulent FMDV strains. Nevertheless, the G–H loops in the Seq 8 fragment were recognized by the antibodies directed against the FMDV strains in both infected pig sera and the commercialized polyclonal antibodies; and given the short sequence of the G–H loop (only 21 residues), it is more likely that the antibodies detected by the Seq 8 antigen to be FMDV-specific neutralizing antibodies directed against the immunodominant epitope of the G–H loop [39, 42]. Moreover, Cao et al. [32] investigated a multi-epitope protein similar to Seq 8 and reported that it has optimal immunogenicity in a mouse model and completely protects against virulent FMDV challenge in pigs [32, 33].

## Conclusions

In the present work, we adopted a simple and reproducible computational approach for the screening of different HEV-FMDV chimeric proteins and accordingly we selected Seq 8-P216 and Seq 8-P222 as the best immunogens. To further confirm the results experimentally, we successfully expressed the selected chimeric antigens in *E. coli* as soluble VLPs, which in turn permitted their purification under native conditions. Further, we found that both proteins were stable at low and high temperatures, especially the Seq 8-P222 protein. Further, this latter has shown optimal antigenicity and immunogenicity properties. To our knowledge, this is the first study to propose the control of HEV infection in the non-human reservoirs (swine) as an attractive and promising approach to prevent the HEV infection in humans. Furthermore, the study presents the first HEV-FMDV chimeric protein (Seq 8-P222) as a combined vaccine candidate: highly expressible in *E. coli*, thermo-stable, self-assembles into VLPs and highly immunogenic.

Finally, the present study provides insights into the vaccine development technology, especially the production of chimeric proteins as vaccine candidates: from the selection of the antigenic regions from the target viruses, to bioinformatics analysis for the selection of the best immunogenic combinations, to plasmids construction and protein expression, and finally to antigenicity and immunogenicity analysis.

## Methods

### Computational analyses

#### Selection of HEV and FMDV immunogens

To select the best antigen candidate to be combined with the FMDV antigen, the HEV ORF2-truncated proteins p166 (aa452–617), p179 (aa439–617), p216 (aa420–637) and p222 (aa439–660) were selected to undergo the bioinformatics analysis and screening. These proteins comprise the dominant neutralizing epitopes of the HEV capsid protein (aa460–605 of the ORF2 protein) [12, 17].

The entire G–H loop domain of VP1 structural protein of O/Mya/98 (Southeast Asia topotype), O/HN/CHA/09 (vaccine strain of Cathay topotype), and O/IRN/2010 (PanAsia 2 topotype) FMDV strains were linked together by two glycine (G) residues according to the order presented in Table 1. Then, the Seq 8 coding DNA was synthesized according to the commonly used codon in *E. coli* (GenScript, Nanjing, China).

**Table 1:** HEV and FMDV individual and combined antigens

N	Sequence description	Sequence ID
	HEV and FMDV individual antigens	
1	G–H loop of O/HN/CHA/09 (Cathay topotype)	Seq-b
2	G–H loop of O/IRN/2010 (PanAsia 2 topotype)	Seq-c
3	G–H loop of O/Mya/98 (Southeast Asia topotype)	Seq-d
4	HEV ORF2 P166 (aa 452–617)	P166
5	HEV ORF2 P179 (aa 439–617)	P179
6	HEV ORF2 P216 (aa 420–637)	P216
7	HEV ORF2 P222 (aa 439–660)	P222
8	(Seq-b)-GG-(Seq-c)-GG-(Seq-d)	Seq8
	HEV-FMDV chimeric antigens	
9	Seq8-HEV ORF2 P166 (aa 452–617)	Seq8-P166
10	Seq8-HEV ORF2 P179 (aa 439–617)	Seq8-P179
11	Seq8-HEV ORF2 P216 (aa 420–637)	Seq8-P216
12	Seq8-HEV ORF2 P222 (aa 439–660)	Seq8-P222
13	HEV ORF2 P166 (aa 452–617)-Seq8	P166-Seq8
14	HEV ORF2 P179 (aa 439–617)-Seq8	P179-Seq8
15	HEV ORF2 P216 (aa 420–637)-Seq8	P216-Seq8
16	HEV ORF2 P222 (aa 439–660)-Seq8	P222-Seq8

#### Design of the HEV-FMDV chimeric antigens

After the selection of the antigens to be screened from HEV (p166, p179, p216, and p222) and FMDV (Seq 8), we next designed a set of chimeric antigens, as shown in Table 1: the FMDV Seq 8 was attached to the N-terminus or C-terminus of the HEV recombinant proteins (p166, p179, p216, and p222), and thus yielded 8 combined proteins. Then, all the designed chimeric antigens were analyzed by bioinformatics tool to select the best immunogenic combinations.

#### Prediction, refinement, and evaluation of the 3D structure models

Phyre2 server was used for protein structure prediction (http://www.sbg.bio.ic.ac.uk/phyre2/html/page.cgi?id=index) [20]. Then, the predicted models were further refined using the GalaxyWeb server (http://galaxy.seoklab.org/index.html) [43]. Finally, the quality of the predicted 3D structures was evaluated using the MolProbity server (http://molprobity.biochem.duke.edu) [44] and the best models were selected for further analysis.

#### Antigenicity analysis by computational methods

To computationally analyze the antigenicity of the chimeric proteins, we have selected different key-epitope sites from both antigens and then analyzed whether combining different HEV and FMDV proteins together could affect the exposition of these key residues and therefore affect the overall antigenicity/immunogenicity of the combined protein.

For HEV antigens, 22 key-epitope sites from the HEV capsid protein were selected namely: E479, Y485, S487, S488, T489, P491, D496, T497, R512, I529, K534, E549, K554, Y561, N562, T564, H577, R578, T585, T586, G591, and P592. These sites have been reported to play a key role in binding different HEV neutralizing antibodies [45].

Concerning FMDV antigens, the most exposed region of the VP1 GH loop is an arginine-glycine-aspartic acid (RGD) tri-peptide that has been reported to be highly conserved in the field strains and mediates cell attachment by binding to integrin receptors [46]. Therefore, the RGD domain of the FMDV Seq 8 antigen in the different chimeric proteins has been analyzed to determine whether its exposition (protrusion) could be affected when it is attached to HEV antigens which may affect the antigenicity and immunogenicity of the FMDV Seq 8 antigen.

After the selection of these key-epitope sites, the antigenicity of all the designed chimeric antigens has been assessed using the ElliPro server (http://tools.immuneepitope.org/ellipro/) [47] and then, we compared the exposition of the preselected key-epitope sites. The ElliPro associates each predicted epitope with a score, defined as a Protrusion Index (PI) value averaged over epitope residues, and this PI varies from 0 (completely buried residues) to 1 (completely exposed residues). As a positive control, the structures of the P2 domain of the HEV capsid protein and the VP1 protein of FMDV type O were retrieved from Protein Data Bank (PDB ID: 2ZTN and 1FOD respectively) and analyzed with Ellipro server.

#### Simulation of the structural flexibility of the selected HEV-FMDV chimeric proteins

Conformation flexibility is a key property of proteins, important for its biological function. Therefore, we sought to analyze the conformational changes that may occur on the structures of the target proteins when they are in an aqueous solution, and whether these changes could affect the exposition of the key-epitope sites. The CABS-flex server (http://biocomp.chem.uw.edu.pl/CABSflex/index.php) has been used to simulate the flexibility of the selected chimeric proteins using the server defaults parameters. The CABS-flex server performs a 10-ns molecular dynamics simulation to obtain a consensus view of protein near-native mobility in solution [48].

Next, the generated models summarizing the mobility profile of the proteins have been submitted to the Ellipro server and the protrusion index of the key epitopes has been calculated and analyzed as described in the previous section (Antigenicity analysis).

### Production of the HEV-FMDV chimeric proteins

#### Amplification of the target genes

The DNA sequences coding for the different HEV target proteins were amplified from the pET28a-P549 plasmid. This plasmid was previously constructed in our lab and it contains the HEV ORF2 coding DNA (HEV genotype 4 strain NJ-703, GenBank No: AY789228). The DNA sequence of the designed FMDV multi-epitope protein (Seq 8) was synthesized by GenScript Incorporation (http://www.genscript.com) according to the most commonly occurring codons in *E. coli*. All the polymerase chain reactions (PCR) were performed using the Expand™ High Fidelity PCR System (Sigma-Aldrich). All the primers are listed in Table 2.

**Table 2:** List of the primers used for the amplification of the different DNA fragments

Primers	5′–3′ sequences
Seq8-NcoI-F	CCC **CCATGG** GGAGCAGCAAGTACGGTGACA
Seq8-XhoI-R	CCC **CTCGAG** AGGCAGCGGCCTCGCCGC
Seq8-SacII-R	CCC **CCGCGG** AGGCAGCGGCCTCGCCGC
P166-SacII-F	CCC **CCGCGG** CCTACCCCCTCCCCT
P166-XhoI-R	CCC **CTCGAG** AGGGTAATCGACAGTGTCC
P216-SacII-F	CCC **CCGCGG** GATAAGGGGATAGCTA
P216-XhoI-R	CCC **CTCGAG** GCCCTGAAGGCCGAGC
P222-SacII-F	CCC **CCGCGG** GTTATCCAGGACTATGATAAT
P222-XhoI-R	CCC **CTCGAG** ATACTCCCGGGTTTTACCCC
pET28a-F	CCCCGCGGATAACAATTCCCCTC
pET28a-R	CCCTCCTTTCGGGCTTTGTTAGCAG

F: forward; R: reverse; the restriction sites are indicated in bold

#### Plasmid construction and transformation of competent *E. coli* cells

The selected HEV and FMDV genes to be combined were modified to contain 5′-NcoI and 3′-SacII restriction sites for the N-terminal fragment (Seq 8) and 5′-SacII and 3′-XhoI restriction sites for the C-terminal fragment (P166, P216, and P222). Then, they were digested with SacII restriction enzyme and ligated with T4 DNA ligase at 16 °C overnight. The linearly ligated genes were further amplified using the 5′-NcoI primer of the N-terminal fragment and the 3′-XhoI primer of the C-terminal fragment.

Next, the combined genes were inserted into the pET28a (+) vector at the NcoI and XhoI restriction sites, downstream of the T7 promoter. All the cloned genes were fused to the pET28a (+) His-tag, which facilitates the detection and purification of the recombinant proteins. The expression constructs were verified by sequence analysis to ensure that all of the genes were inserted correctly, and used to transform competent *E. coli* (BL21) cells. Finally, the grown clones were verified for the presence of the target genes using PCR and DNA sequencing.

#### Protein expression and purification

For each protein, a single colony was picked and grown overnight at 37 °C in Luria–Bertani broth (LB) containing 50 μg/ml kanamycin (LB/Kan+) as a starter culture. The overnight culture was diluted 1:100 in 200 ml of LB/Kan+ and grown to an OD_600_ between 0.6 and 0.7. The expression was induced for 3 h by the addition of IPTG to a final concentration of 0.5 mM. To verify the expression of the target proteins, the cells were collected by centrifugation and the pellet was analyzed by 15% sodium dodecyl sulfate–polyacrylamide gel electrophoresis (SDS–PAGE). For large scale purification, cultures were harvested by centrifugation in a Beckman Allegra™ 21R centrifuge at 6000 rpm at 4 °C for 20 min. Cell pellets were stored at − 80 °C.

To analyze the solubility of the expressed proteins, the bacteria pellets harvested after the induction of the expression were resuspended in a lysis buffer (50 mM NaH_2_PO_4_, pH 8.0, containing 300 mM NaCl and 10 mM imidazole). After three freeze/thaw cycles, the cells were treated with lysozyme (added to a final concentration of 0.5 mg/ml) and incubated on ice for 2 h. Then, deoxyribonuclease (DNase) was added to a final concentration of 25 U/ml to remove the viscosity caused by the DNA content in bacterial cell lysates. The suspensions were then clarified by centrifugation (10 min at 12,000×*g* and 4 °C) and aliquots of both pellets and supernatants were analyzed by 12% SDS-PAGE or kept at − 80 °C until further analysis.

Finally, since all the expressed proteins were C-terminally His-tagged, the soluble fractions of the proteins were purified under native conditions using Ni–NTA affinity chromatography as described previously [27].

#### Stability analysis of the expressed proteins

The purified proteins were pooled and stored at different temperatures: 37 °C, 4 °C, − 20 °C and − 80 °C. Then, the degradation rate was analyzed by SDS-PAGE at different time points: after 2 weeks, 6 weeks and 10 weeks.

#### Analysis of VLP formation by transmission electron microscopy

The target proteins (0.1 g/ml) were adsorbed onto carbon-coated grids for 10 min, then negatively stained with 2% uranyl acetate for 30 min, and examined under a transmission electron microscope (F30, Philips, The Netherlands).

### Assessment of the antigenicity/immunogenicity of the HEV-FMDV chimeric proteins

#### Western blotting

The purified proteins Seq 8, Seq 8-P216, Seq 8-P222 and P222 were separated by SDS-PAGE using 12% polyacrylamide gel. Then, the proteins were electrotransferred onto nitrocellulose membranes (200 mA for 1.5 h). After completing the transfer, the membranes were removed and placed in the blocking solution (5% non-fat milk in 20 mM Tris and 0.9% NaCl Tris-buffered saline, pH: 7.6, supplemented with 0.1% Tween 20) and incubated for 2 h at 4 °C. Then, the membranes were incubated overnight with the primary antibody diluted in the blocking buffer (commercial porcine polyclonal antibodies used against FMD diluted 1:500 or anti-HEV 5G5 monoclonal antibody diluted 1:1000). The next day, the blots were washed and incubated with agitation for 2 h at room temperature with the secondary antibody solution (Goat anti-mouse or anti-pig IgGs conjugated with horseradish-peroxidase) diluted in the blocking buffer. Finally, the membranes were developed with the DAB-Peroxidase substrate solution.

#### Mice immunization

A total of 60 female BALB/c mice (6–8 weeks-old) were purchased from the Comparative Medicine Center of Yangzhou University. Prior to any experiment, the mice were tested and found free of anti-HEV and anti-FMDV antibodies. Animal experiments were performed according to the guidelines of the Chinese National Science and Technology Commission for animal experimentation and approved by the Institutional Animal Care and Use Committee of Southeast University.

The animals were randomly divided into 6 groups (10 mice/group) and were allowed free access to food and water. The experimental groups were separately immunized by intramuscular injection of 0.1 ml of immunization solutions containing respectively: 50 µg of purified Seq 8, Seq-P216 or Seq 8-P222; 10 µg of purified P222; 100 µl of commercially available FMD inactivated vaccine; 100 µl of sterile saline solution. All the immunization solutions were prepared with the MONTANIDE ISA-206 adjuvant (China Agricultural Vet. Bio. Science and Technology Co., Lanzhou, China) at an adjuvant: antigen ratio of 1:1. The animals were immunized on days 0 and 15. The blood samples were collected on weeks 2, 4, 6, 8 and 12 after the first immunization and the sera were isolated and stored at − 80 °C until analysis.

#### Indirect enzyme-linked immunosorbent assay (ELISA)

The indirect ELISA was adopted to determine the antigenicity of the expressed proteins and to detect the presence of anti-HEV and/or anti-FMDV antibodies in the sera of the immunized mice.

The procedure was executed as follows: the coating antigen was diluted in coating buffer (15 mM Na_2_CO_3_ and 35 mM NaHCO_3_, pH: 9.5) to a final concentration of 1 µg/ml and 100 µl of the dilution were added to each well of 96-well flat-bottomed plate (100 ng/well). Then, the plates were incubated for 2 h at 37 °C. Next, the plates were washed three times with 200 µl/well phosphate-buffered saline (PBS: 137 mM NaCl, 2.7 mM KCl, 10 mM Na_2_HPO_4_, 1.8 mM KH_2_PO_4_, pH: 7.4) supplemented with 0.05% Tween 20. Test and control sera serial dilutions in 1% Casein PBS (1: 200, 1: 400, 1: 800, 1: 1600, 1: 3200, 1: 6400, 1:12,800 and 1:25,600) were distributed into the wells (100 µl/well) and incubated for 1 h at 37 °C. Next, the primary antibody solution was removed and the plates were washed four times PBS-0.05% Tween 20 (200 µl/well). The HRP-conjugated goat anti-mouse or anti-pig IgG was diluted in 1% Casein PBS 1:2000, according to the manufacturer instructions and 100 µl of the dilution was added to each well. After incubation at 37 °C for 1 h, the plates were washed four times and the reaction was detected by adding 100 µl/well of 1:1 peroxidase solution/TMB substrate solution. Finally, after 30 min at 37 °C, the reaction was stopped by adding 50 µl/well of 2 M H_2_SO_4_ and the plates were read using a kinetic microplate reader at a wavelength of 450 nm.

First, the chimeric proteins Seq 8-P216 and Seq 8-P222, as well as Seq 8 and P222, were used as coating antigens; and a set of serum samples was used to determine whether the chimeric proteins could react against the anti-HEV and anti-FMDV antibodies. The set comprised sera of pigs infected with Mya98 and Cathy FMDV strains, sera of pigs free of HEV and FMDV infections (as a negative control) and finally the anti-HEV neutralizing 5G5 monoclonal antibody.

Further, the sera collected from the immunized mice at different time points after the first injection were assessed for the presence of anti-HEV and anti-FMDV specific antibodies using the above described indirect ELISA procedure, where P166 and Seq 8 proteins were used as the coating antigens.

#### Statistical analysis

All the graphs were prepared using GraphPad 5 software (GraphPad Software, Inc., San Diego, CA). The indirect ELISA results are presented as mean ± SEM (standard error of the mean). Two-way ANOVA followed by Tukey’s test was used to compare the levels of the elicited anti-HEV and anti-FMDV antibodies between the different groups. P < 0.05 was used to indicate statistical significance.

## Supplementary information

**Additional file 1.** Predicted 3D structure models of the HEV and FMDV individual antigens. Structures are shown as cartoon representations where the HEV antigens (P166, P179, P216 and P222) are depicted in cyan and the FMDV antigen (Seq 8) is depicted in yellow. Structure-related figures were prepared using the program PyMol.

**Additional file 2.** Predicted 3D structure models of the HEV-FMDV recombinant chimeric proteins. Structures are shown as cartoon representations where the HEV fragments are depicted in cyan and the FMDV fragment is depicted in yellow. Structure-related figures were prepared using the program PyMol.

**Additional file 3.** Clustering data of Seq8-P216 and Seq8-P222 conformation models (Table S1) and fluctuations between the different Seq8-P216 and Seq8-P222 conformation clusters (Table S2) obtained in the flexibility analysis.

**Additional file 4.** Detection of anti-FMDV antibodies in the serial dilutions of sera of mice immunized with Seq 8, Seq 8-P216, Seq 8-P222 and FMDV commercially available vaccine, at different time points post inoculation, using indirect ELISA.

**Additional file 5.** Detection of anti-HEV antibodies in the serial dilutions of sera of mice immunized with Seq 8, Seq 8-P216, Seq 8-P222 and P222, at different time points post inoculation, using indirect ELISA.

## Data Availability

All computational data generated, analyzed and reported in the present work as well as the materials used are available from the corresponding author upon request.
