# Supplementary figures and images for: Design and development of a chimeric vaccine candidate against zoonotic hepatitis E and foot-and-mouth disease
Source: Microb Cell Fact. 2020 Jul 11;19:137. doi: 10.1186/s12934-020-01394-1 (PMC7352093; doi:10.1186/s12934-020-01394-1)

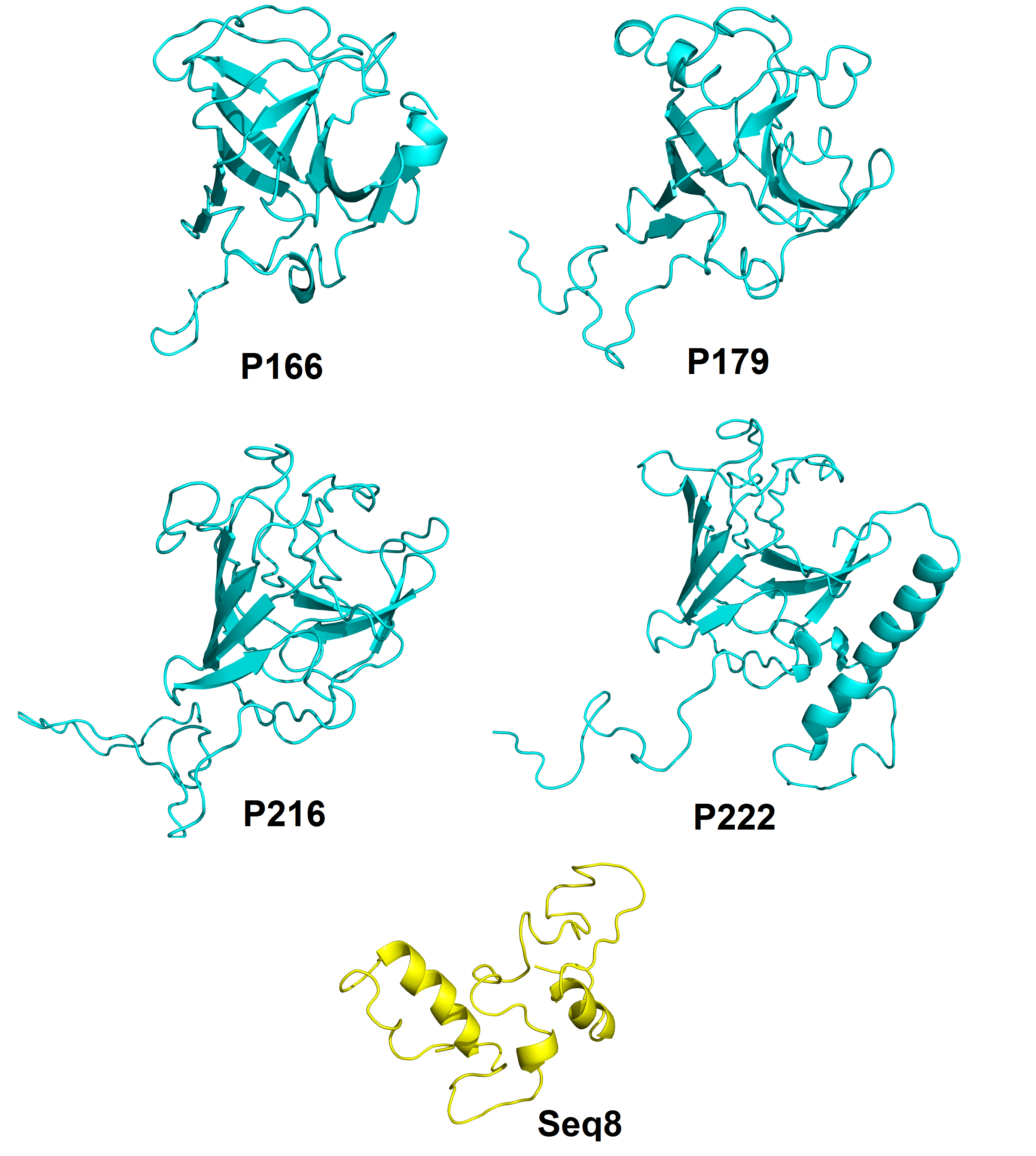

Supplement: Supplementary file 1 — Additional file 1. Predicted 3D structure models of the HEV and FMDV individual antigens. Structures are shown as cartoon representations where the HEV antigens (P166, P179, P216 and P222) are depicted in cyan and the FMDV antigen (Seq 8) is depicted in yellow. Structure-related figures were prepared using the program PyMol. [file 12934_2020_1394_MOESM1_ESM.tif]

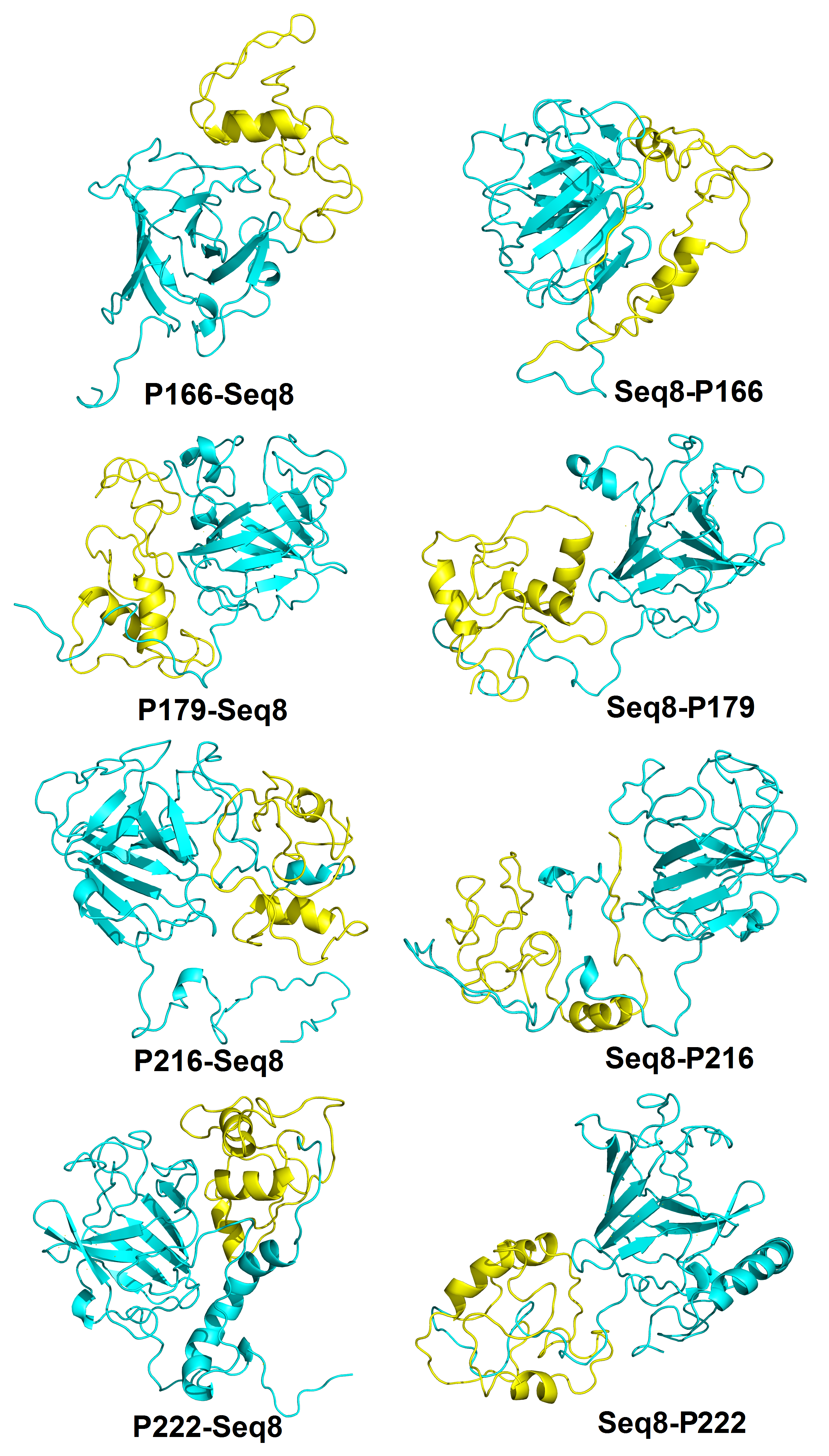

Supplement: Supplementary file 2 — Additional file 2. Predicted 3D structure models of the HEV-FMDV recombinant chimeric proteins. Structures are shown as cartoon representations where the HEV fragments are depicted in cyan and the FMDV fragment is depicted in yellow. Structure-related figures were prepared using the program PyMol. [file 12934_2020_1394_MOESM2_ESM.tif]

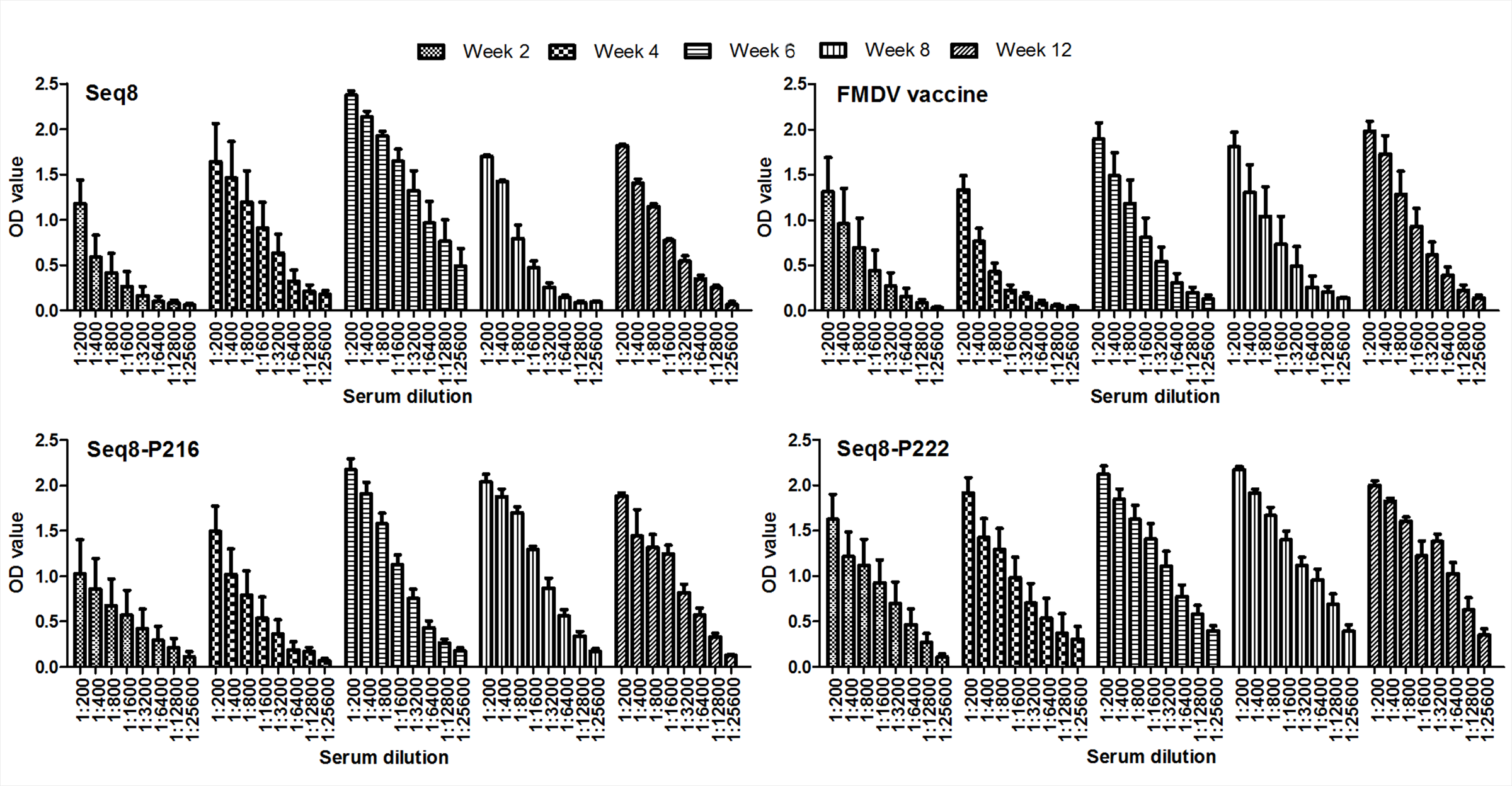

Supplement: Supplementary file 4 — Additional file 4. Detection of anti-FMDV antibodies in the serial dilutions of sera of mice immunized with Seq 8, Seq 8-P216, Seq 8-P222 and FMDV commercially available vaccine, at different time points post inoculation, using indirect ELISA. [file 12934_2020_1394_MOESM4_ESM.tif]

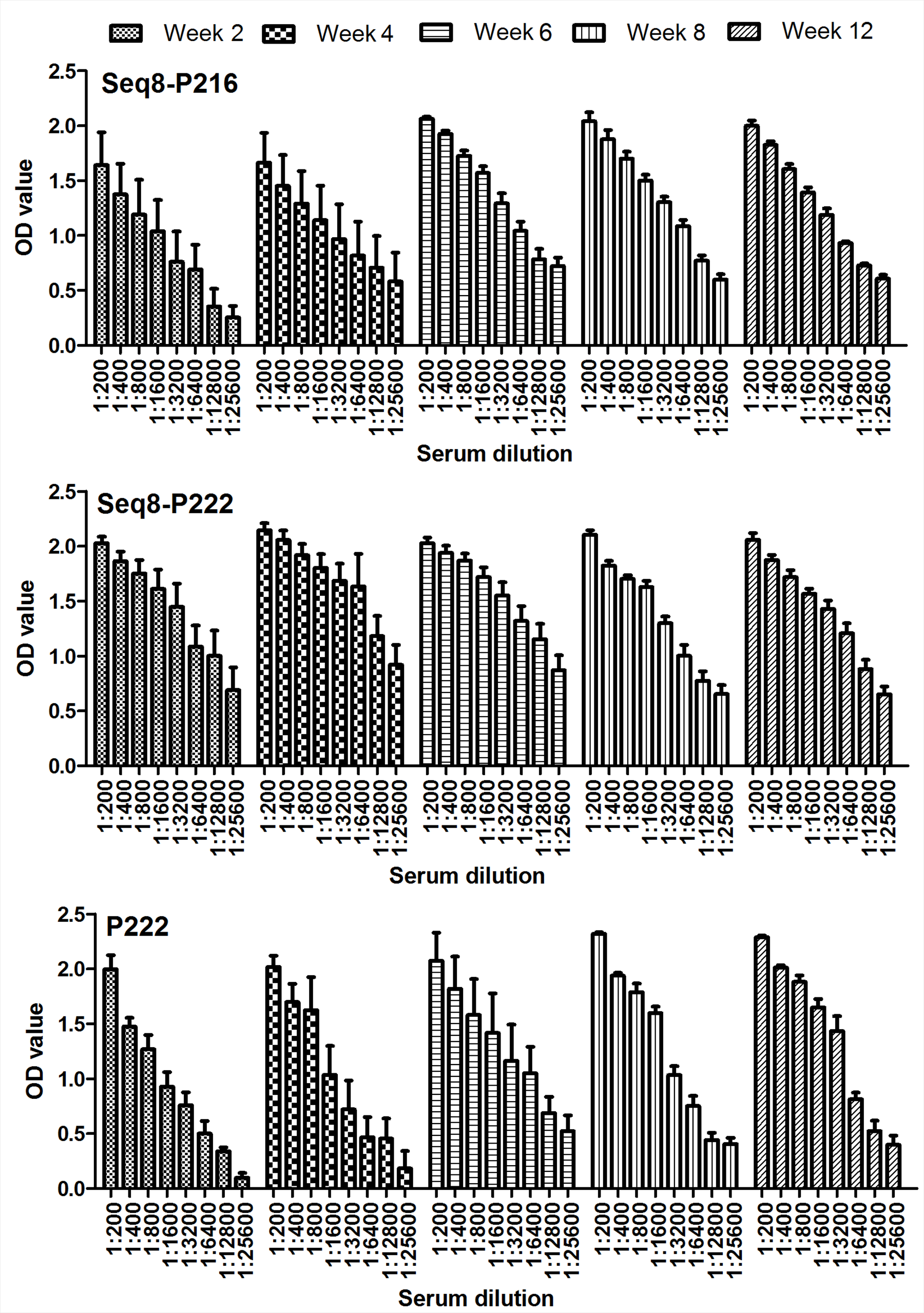

Supplement: Supplementary file 5 — Additional file 5. Detection of anti-HEV antibodies in the serial dilutions of sera of mice immunized with Seq 8, Seq 8-P216, Seq 8-P222 and P222, at different time points post inoculation, using indirect ELISA. [file 12934_2020_1394_MOESM5_ESM.tif]
