# Supplementary material for: Design and development of a chimeric vaccine candidate against zoonotic hepatitis E and foot-and-mouth disease
Source: Microb Cell Fact. 2020 Jul 11;19:137. doi: 10.1186/s12934-020-01394-1 (PMC7352093; doi:10.1186/s12934-020-01394-1)
Supplement: Supplementary file 3 — Additional file 3. Clustering data of Seq8-P216 and Seq8-P222 conformation models (Table S1) and fluctuations between the different Seq8-P216 and Seq8-P222 conformation clusters (Table S2) obtained in the flexibility analysis. [file 12934_2020_1394_MOESM3_ESM.docx]

**Table S1. Clustering data of Seq8-P216 and Seq8-P222 conformation models**

|  | **Seq8-P216** | | |  | **Seq8-P222** | | |
| --- | --- | --- | --- | --- | --- | --- | --- |
| **Cluster** | **Cluster density** | **Cluster size** | **Average cluster RMSD** |  | **Cluster density** | **Cluster size** | **Average cluster RMSD** |
| 1 | 149.9 | 300 | 2 |  | 155.8 | 285 | 1.8 |
| 2 | 114.6 | 260 | 2.3 |  | 136.5 | 243 | 1.8 |
| 3 | 94.9 | 189 | 2 |  | 111.5 | 212 | 1.9 |
| 4 | 93.3 | 187 | 2 |  | 105.6 | 197 | 1.9 |
| 5 | 92.7 | 201 | 2.2 |  | 103.5 | 182 | 1.8 |
| 6 | 75.8 | 137 | 1.8 |  | 81.4 | 134 | 1.6 |
| 7 | 75.7 | 160 | 2.1 |  | 78 | 134 | 1.7 |
| 8 | 65.7 | 139 | 2.1 |  | 77.6 | 145 | 1.9 |
| 9 | 58.5 | 112 | 1.9 |  | 77 | 140 | 1.8 |
| 10 | 58.5 | 117 | 2 |  | 72.3 | 133 | 1.8 |
| 11 | 51.1 | 102 | 2 |  | 55.5 | 91 | 1.6 |
| 12 | 50.8 | 96 | 1.9 |  | 53.7 | 104 | 1.9 |

^Cluster density= cluster size divided by average cluster RMSD, rounded to one decimal place;^

^RMSD: root-mean-square deviation^

**Table S2. Fluctuations between the different Seq8-P216 and Seq8-P222 conformation clusters**

| **Cα GDT-TS between predicted models** | | | | | | | | | | | | | |
| --- | --- | --- | --- | --- | --- | --- | --- | --- | --- | --- | --- | --- | --- |
|  |  | **Seq8-P216** | | | | | | | | | | | |
|  |  | 1 | 2 | 3 | 4 | 5 | 6 | 7 | 8 | 9 | 10 | 11 | 12 |
| **Seq8-P222** | 1 |  | 0.56 | 0.56 | 0.62 | 0.53 | 0.6 | 0.57 | 0.52 | 0.52 | 0.51 | 0.58 | 0.55 |
|  | 2 | 0.54 |  | 0.52 | 0.57 | 0.57 | 0.54 | 0.59 | 0.5 | 0.48 | 0.51 | 0.59 | 0.49 |
|  | 3 | 0.59 | 0.56 |  | 0.58 | 0.51 | 0.56 | 0.51 | 0.51 | 0.56 | 0.46 | 0.52 | 0.58 |
|  | 4 | 0.57 | 0.59 | 0.59 |  | 0.52 | 0.61 | 0.56 | 0.53 | 0.52 | 0.49 | 0.57 | 0.56 |
|  | 5 | 0.5 | 0.6 | 0.56 | 0.57 |  | 0.53 | 0.55 | 0.53 | 0.47 | 0.55 | 0.5 | 0.49 |
|  | 6 | 0.5 | 0.64 | 0.55 | 0.6 | 0.67 |  | 0.54 | 0.49 | 0.54 | 0.48 | 0.52 | 0.56 |
|  | 7 | 0.59 | 0.66 | 0.57 | 0.63 | 0.62 | 0.62 |  | 0.5 | 0.51 | 0.48 | 0.54 | 0.52 |
|  | 8 | 0.53 | 0.69 | 0.57 | 0.58 | 0.64 | 0.67 | 0.63 |  | 0.45 | 0.58 | 0.54 | 0.5 |
|  | 9 | 0.54 | 0.56 | 0.59 | 0.65 | 0.58 | 0.58 | 0.59 | 0.59 |  | 0.44 | 0.48 | 0.6 |
|  | 10 | 0.6 | 0.52 | 0.58 | 0.56 | 0.54 | 0.52 | 0.54 | 0.54 | 0.54 |  | 0.5 | 0.47 |
|  | 11 | 0.56 | 0.6 | 0.63 | 0.6 | 0.63 | 0.59 | 0.63 | 0.59 | 0.65 | 0.56 |  | 0.48 |
|  | 12 | 0.56 | 0.51 | 0.55 | 0.58 | 0.49 | 0.49 | 0.52 | 0.5 | 0.53 | 0.6 | 0.56 |  |
| **Cα RMSD between predicted models** | | | | | | | | | | | | | |
|  |  | **Seq8-P216** | | | | | | | | | | | |
|  |  | 1 | 2 | 3 | 4 | 5 | 6 | 7 | 8 | 9 | 10 | 11 | 12 |
| **Seq8-P222** | 1 |  | 4.26 | 3.81 | 3.12 | 4.58 | 3.2 | 3.84 | 4.52 | 4.54 | 4.92 | 3.49 | 4.12 |
|  | 2 | 4.21 |  | 5.38 | 4.28 | 3.91 | 4.8 | 3.4 | 4.86 | 6.29 | 4.94 | 3.71 | 6.03 |
|  | 3 | 3.59 | 3.81 |  | 3.62 | 4.87 | 3.58 | 4.71 | 5.14 | 3.68 | 5.9 | 4.48 | 3.55 |
|  | 4 | 4.01 | 3.96 | 3.93 |  | 4.5 | 3.14 | 3.96 | 4.37 | 4.9 | 5.01 | 3.45 | 4.01 |
|  | 5 | 4.44 | 3.04 | 3.7 | 4.09 |  | 4.32 | 4.34 | 4.82 | 5.71 | 4.15 | 4.86 | 5.33 |
|  | 6 | 4.94 | 3.37 | 4.05 | 4.33 | 2.66 |  | 4.26 | 5.24 | 4.27 | 5.4 | 4.38 | 3.76 |
|  | 7 | 3.68 | 2.88 | 3.66 | 3.53 | 3.1 | 3.27 |  | 5.04 | 5.59 | 5.54 | 3.99 | 5.38 |
|  | 8 | 4.44 | 2.5 | 3.81 | 4.28 | 3.05 | 3.18 | 3.09 |  | 6.29 | 3.79 | 4.28 | 5.45 |
|  | 9 | 4.32 | 3.98 | 3.7 | 3.05 | 3.93 | 4.06 | 3.53 | 4.05 |  | 6.48 | 6.01 | 3.43 |
|  | 10 | 3.45 | 4.49 | 3.93 | 3.84 | 4.86 | 5.06 | 4.18 | 4.55 | 4.31 |  | 5.08 | 5.73 |
|  | 11 | 3.87 | 3.58 | 3.04 | 3.57 | 3.16 | 3.44 | 3.16 | 3.46 | 3.13 | 4.13 |  | 5.43 |
|  | 12 | 4.12 | 5.12 | 4.51 | 4.09 | 5.57 | 5.78 | 4.62 | 5.35 | 4.36 | 3.42 | 4.61 |  |

^The fluctuations between the different clusters of Seq8-P216 and Seq8-P222 conformations were calculated in terms of Cα RMSD and Cα GDT-TS (Global Distance Test-Total Score). The upper and bottom triangles represent the results for Seq8-P216 and Seq8-P222, respectively. The table is presented as a heat map to better interpret the results: 1) For the Cα GDT-TS, the color varies from 0.4 as a minimal value (white) to 0.7 as the maximal value (red); higher GDT score indicates more similarity between the models. 2) For the Cα RMSD, the color varies from 4 as a minimal value (white) to 8 as a maximal value (blue); higher RMSD score indicates more dissimilarity between the models.^
